# Assessment of MXD3 Expression as a Predictor of Survival in Lung Squamous Cell Carcinoma

**DOI:** 10.1155/ijog/7355595

**Published:** 2025-05-15

**Authors:** Mingzhi Cao, Ning Zhang, Tangbing Chen, Hong Jiang

**Affiliations:** Department of Thoracic Surgery, 905th Hospital of People's Liberation Army Navy, Naval Medical University, Shanghai, China

**Keywords:** biomarkers, lung squamous cell carcinoma, prognosis, survival analysis

## Abstract

**Backgrounds and Aims:** Lung squamous cell carcinoma (LUSC) represents a significant challenge in oncology, necessitating the identification of novel prognostic markers and therapeutic targets. This study is aimed at investigating the oncogenic role of MXD3 (MAX Dimerization Protein 3) in LUSC and its implications for patient prognosis.

**Methods:** A retrospective cohort of 199 LUSC patients from the 905th Hospital of People's Liberation Army Navy was analyzed to evaluate MXD3 expression levels and their association with clinicopathological characteristics and survival outcomes. Immunohistochemistry (IHC) staining was performed to assess MXD3 expression in LUSC tissue samples. Survival analyses, including the Kaplan–Meier curves and multivariate Cox regression, were conducted to determine the prognostic significance of MXD3 expression and other clinicopathological factors. Additionally, the methylation status of MXD3 was examined using data from the TCGA database to assess its role in regulating MXD3 expression and survival outcomes.

**Results:** MXD3 expression exhibited significant heterogeneity among LUSC patients, with high MXD3 expression correlating with advanced tumor differentiation grade, larger tumor size, and advanced T and N stages. The Kaplan–Meier survival analyses revealed that high MXD3 expression was associated with shorter cancer-specific survival. Multivariate Cox regression identified MXD3 expression level and lymph node involvement (N stage) as independent prognostic factors for cancer-specific survival in LUSC patients. Additionally, analysis of MXD3 methylation revealed significantly lower methylation levels in LUSC tissues, and reduced methylation correlated with poorer survival outcomes.

**Conclusions:** Our findings highlight MXD3 as a promising prognostic biomarker for LUSC, with high MXD3 expression predicting poorer survival outcomes. MXD3 expression level, along with lymph node involvement and methylation status, could serve as independent prognostic indicators for risk stratification and treatment decision-making in LUSC patients. Further research is warranted to elucidate the underlying mechanisms of MXD3-mediated tumorigenesis and its potential as a therapeutic target in LUSC management.

## 1. Introduction

Lung squamous cell carcinoma (LUSC) is a major subtype of non-small cell lung cancer (NSCLC) and presents significant clinical challenges due to its aggressive nature, poor prognosis, and limited treatment options [1, 2]. Despite advancements in early detection and treatment, the survival rate for LUSC patients remains low, with the overall prognosis being unfavorable due to factors such as late-stage diagnosis and resistance to conventional therapies [3].

The molecular landscape of LUSC has been increasingly explored through genomic and transcriptomic studies, revealing a number of potential biomarkers and therapeutic targets. Recent research has highlighted several key genes and signaling pathways involved in LUSC pathogenesis, including those regulating cell proliferation, apoptosis, and metastasis [4–6]. Among these, MXD3 (MAX Dimerization Protein 3), a member of the MYC/MAX/MXD network, has garnered attention as a potentially significant player in tumorigenesis. MXD3 regulates critical cellular processes such as growth, differentiation, and apoptosis [7, 8], and its dysregulation has been implicated in a range of cancers, including glioma and leukemia [9, 10].

The specific role of MXD3 in LUSC, however, remains poorly characterized, and few studies have explored its potential as a prognostic marker or therapeutic target in this malignancy. Notably, recent evidence suggests that dysregulation of the MYC/MAX/MXD network contributes to the progression of multiple cancers, highlighting MXD3 as a gene of interest for further investigation in the context of LUSC. This study is aimed at addressing this gap by examining the expression and functional implications of MXD3 in LUSC, seeking to elucidate its role in tumor progression and its potential utility as a clinical biomarker.

We hypothesize that alterations in MXD3 expression contribute to the aggressive nature of LUSC and that MXD3 could serve as a prognostic biomarker for patient outcomes. To test this hypothesis, we analyze MXD3 expression patterns in LUSC tissue samples and correlate these with clinical and pathological features, including tumor differentiation, stage, and survival data. Additionally, we explore the potential therapeutic implications of targeting MXD3, given its role in the MYC pathway, which is known to be involved in cancer cell proliferation and survival. By investigating MXD3's molecular and clinical significance in LUSC, this study not only adds to the growing body of knowledge surrounding the disease but also offers insights into new avenues for therapeutic intervention and personalized treatment strategies for patients with LUSC.

## 2. Methods

### 2.1. Cohort Enrollment

A retrospective cohort of patients diagnosed with LUSC was enrolled from the 905th Hospital of People's Liberation Army Navy. Inclusion criteria encompassed patients with histologically confirmed LUSC, availability of clinical and pathological data, and sufficient follow-up information. Patients with incomplete medical records or who underwent neoadjuvant therapy were excluded from the study. In addition, patients who survived less than 6 months were also excluded to avoid potential bias related to early mortality events. Clinical and pathological data, including age, sex, tumor differentiation grade, tumor site, laterality, tumor size, T stage, and N stage, were collected from electronic medical records and pathology reports. Tumor samples were obtained from surgical resections or biopsy specimens, and histological diagnosis was confirmed by experienced pathologists.

### 2.2. Survival Analyses and Statistics

Survival analyses were conducted to assess cancer-specific survival outcomes in the enrolled LUSC cohort. The Kaplan–Meier survival curves were constructed to visualize survival probabilities based on various clinicopathological factors, including age, sex, differentiation grade, tumor site, laterality, tumor size, T stage, and N stage. The log-rank test was employed to compare survival curves and assess the significance of differences in survival outcomes. Additionally, multivariate Cox regression analysis was performed to identify independent prognostic factors for cancer-specific survival in LUSC patients. Hazard ratios (HRs) and 95% confidence intervals (CIs) were calculated to estimate the relative risk of cancer-related mortality associated with each prognostic factor. Statistical analyses were conducted using R Software, and *p* values less than 0.05 were considered statistically significant.

### 2.3. Extraction and Analysis of TCGA Data

Clinical, genomic, and methylation data were accessed through the Genomic Data Commons (GDC) Portal (https://portal.gdc.cancer.gov/), which provides comprehensive datasets from multiple cancer types, including LUSC. The LUSC cohort from TCGA was used in this study, which provided clinical data (e.g., survival and tumor characteristics) as well as genomic and methylation information for the patients.

To investigate the methylation status of MXD3, we retrieved DNA methylation data corresponding to CpG sites across the genome. The methylation data were normalized and processed using the minfi package in R, which includes quality control steps to ensure data integrity. The beta values from specific CpG sites were used to assess the relationship between methylation and MXD3 expression levels. Statistical tests were performed to explore the correlation between methylation status and MXD3 expression, as well as to evaluate the impact of these factors on survival outcomes in the LUSC cohort.

### 2.4. Immunohistochemistry (IHC)

IHC analysis was performed to assess MXD3 protein expression in tumor tissue samples. Formalin-fixed, paraffin-embedded (FFPE) tissue sections were deparaffinized and subjected to antigen retrieval. The slides were incubated with a primary antibody against MXD3 (Abcam, Cat#. ab108525; 1:1000 dilution) for 12 h, followed by incubation with a secondary antibody conjugated to a detection system. The IHC results were evaluated by two independent pathologists. Protein expression levels were quantified based on staining intensity and the percentage of positive cells, and the final expression score was used for statistical analysis.

### 2.5. Ethics

This study was conducted in accordance with the principles outlined in the Declaration of Helsinki and was approved by the Institutional Review Board (IRB) of the 905th Hospital of People's Liberation Army Navy (No. PLA905YY230067). Patient data were anonymized and handled confidentially to ensure privacy protection. Informed consent was obtained from all participants or, in cases where patients were unable to provide consent (e.g., due to age, mental incapacity, or other reasons), from their legal representatives or direct relatives, in accordance with local ethical guidelines and regulatory requirements. This practice is in line with the ethical standards for retrospective studies in our region, where the use of anonymized or deidentified patient data is common, and consent from direct relatives is permissible when patients cannot provide consent themselves. All research activities adhered to the relevant ethical guidelines and regulations.

## 3. Results

### 3.1. Expression Difference of MXD3 in LUSC

**Figure 1:**
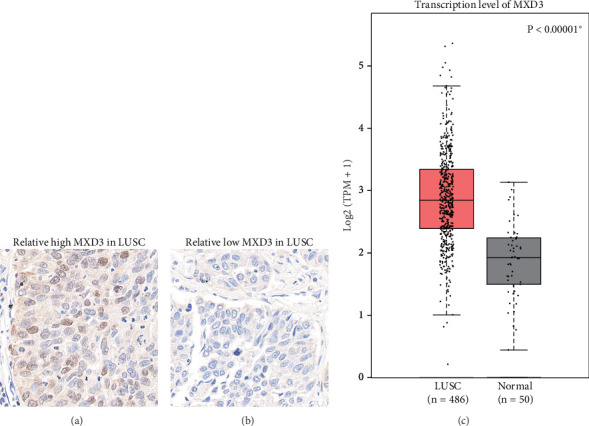
Expression difference of MXD3 in LUSC. (a) Representative high MXD3 expression in LUSC tissues as observed through immunohistochemistry (IHC) staining. (b) Representative low MXD3 expression in LUSC tissues as observed through IHC staining. (c) Difference of transcriptional level of MXD3 in TCGA dataset, showing a significantly higher MXD3-mRNA level in LUSC tissues than normal lung tissues.

IHC staining was employed to assess the expression levels of MXD3 in LUSC tissues.  Figure 1a illustrates a representative image showing high MXD3 expression in LUSC tissue samples. Intense immunostaining is observed throughout the tissue sections, indicating elevated levels of MXD3 protein in the tumor cells. Conversely, Figure 1b depicts a representative image displaying low MXD3 expression in LUSC tissues. In contrast to the high expression group, diminished immunoreactivity for MXD3 is observed in these tissue samples, suggestive of relatively low MXD3 protein levels. These findings highlight the heterogeneity in MXD3 expression among LUSC patients, with some exhibiting robust MXD3 expression while others demonstrate attenuated levels. Interestingly, we further retrieved the mRNA level of MXD3 in LUSC tissues in the TCGA dataset, which can help further validate our findings in our small retrospective cohort. As a result, the TCGA data showed a significantly higher MXD3-mRNA level in LUSC tissues than in normal lung tissues (Figure 1c, *p* < 0.00001).

### 3.2. Clinicopathological Correlations of MXD3 Expression in LUSC Patients

Table 1 summarizes the clinicopathological characteristics of the enrolled LUSC cases and their associations with MXD3 expression levels. A retrospective cohort comprising 199 LUSC patients from our hospital was analyzed to investigate the correlations between MXD3 expression and patients' characteristics. Among the parameters, the age of 60 years old and tumor size of 3.0 cm were selected as the cut-off values, based on common clinical practice and guidelines. The cut-off value of MXD3 expression was based on the median IHC staining score. There was no significant association between MXD3 expression and age (*p* = 0.716) or sex (*p* = 0.726) of the patients. However, MXD3 expression exhibited a statistically significant association with tumor differentiation grade (⁣^∗^*p* = 0.023), tumor size (⁣^∗^*p* = 0.006), T stage (⁣^∗^*p* = 0.004), and N stage (⁣^∗^*p* < 0.001). Notably, MXD3 expression levels were found to correlate with tumor differentiation grade, with higher MXD3 expression observed in poorly differentiated tumors compared to well and moderately differentiated tumors. Similarly, MXD3 expression was significantly associated with larger tumor size (≥ 3.0 cm) and advanced T and N stages, suggesting a potential role of MXD3 in tumor aggressiveness and metastasis in LUSC. Consistently, a higher rate of high MXD3 was observed in patients who underwent postoperative chemotherapy (*p* = 0.025) or radiotherapy (*p* = 0.013). These findings underscore the clinical relevance of MXD3 expression in LUSC and its potential utility as a prognostic marker for disease progression and patient outcomes.

### 3.3. Univariate Survival Analyses in LUSC Patients

**Figure 2:**
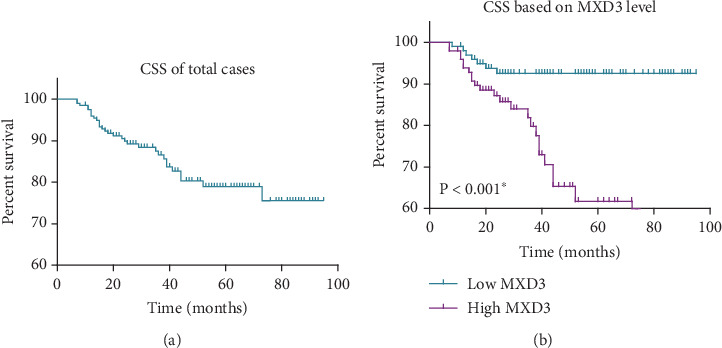
Kaplan–Meier survival curves to assess the prognostic role of MXD3 in LUSC cohort. (a) Kaplan–Meier analysis of cancer-specific survival in the entire cohort of LUSC patients. (b) Kaplan–Meier analysis of cancer-specific survival in LUSC cases stratified by MXD3 expression levels.

To further investigate the prognostic significance of various clinicopathological factors, we conducted univariate survival analyses for cancer-specific survival in LUSC patients (Figure 2a, Table 2), with a focus on various clinicopathological variables including age, sex, differentiation grade, tumor site, laterality, tumor size, T stage, N stage, and MXD3 expression levels. Overall, the mean cancer-specific survival time and 5-year survival rates were calculated for each variable to assess their prognostic significance.

Notably, MXD3 expression levels exhibited a profound impact on cancer-specific survival in LUSC patients (Figure 2b), ⁣^∗^*p* < 0.001). Patients with low MXD3 expression demonstrated significantly longer mean survival time (89.1 ± 2.1 months) and a higher 5-year survival rate (92.5%) compared to those with high MXD3 expression levels (mean survival time: 64.9 ± 3.9 months, 5-year survival rate: 61.7%), highlighting MXD3 as a potential prognostic biomarker for LUSC. These findings underscore the importance of MXD3 expression as a prognostic indicator in LUSC, suggesting its potential utility in risk stratification and treatment decision-making for LUSC patients.

**Figure 3:**
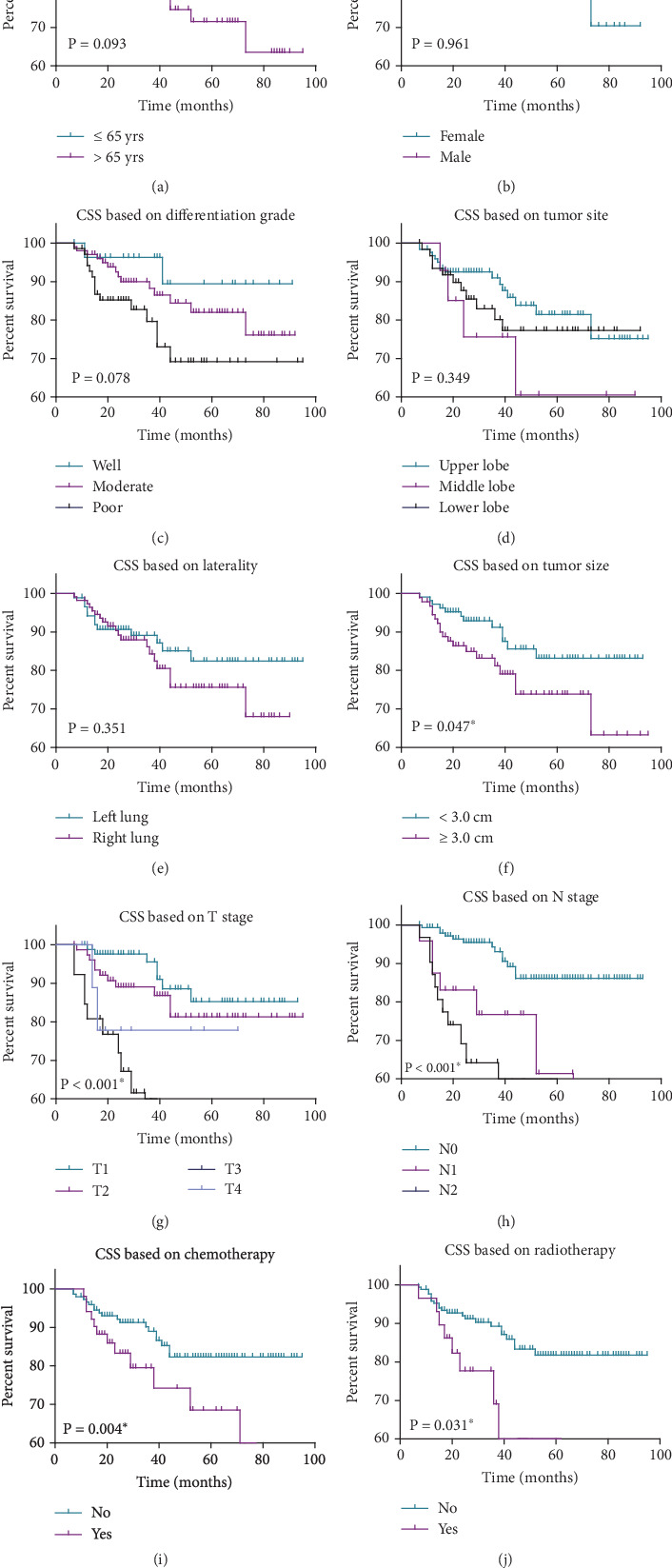
Kaplan–Meier cancer-specific survival curves to assess the prognosis of enrolled LUSC cases. Kaplan–Meier survival curves illustrating cancer-specific survival outcomes in enrolled LUSC cases. The survival curves were constructed based on patients' characteristics: (a) age, (b) sex, (c) differentiation grade, (d) tumor site, (e) laterality, (f) tumor size, (g) T stage, (h) N stage. (i) radiotherapy, and (j) chemotherapy. The curves were compared using the log-rank test to assess statistical significance.

As for other factors (Figure 3a, 3b, 3c, 3d, 3e, 3f, 3g, and 3h), age at diagnosis demonstrated a trend towards significance (*p* = 0.093), with patients aged ≤ 65 years exhibiting a slightly longer mean survival time (82.5 ± 2.7 months) and higher 5-year survival rate (84.7%) compared to those aged > 65 years (74.6 ± 4.2 months, 71.5%). However, this difference did not reach statistical significance (*p* = 0.093). Similarly, sex, differentiation grade, tumor site, and laterality did not show significant associations with cancer-specific survival (*p* > 0.05). However, tumor size (⁣^∗^*p* = 0.047), T stage (⁣^∗^*p* < 0.001), and N stage (⁣^∗^*p* < 0.001) emerged as significant prognostic factors, with larger tumor size (74.4 ± 4.3 vs. 82.6 ± 2.8 months), advanced T and N stages correlating with poorer survival outcomes.

Furthermore, considering that postoperative adjuvant therapy may also contribute to the distinct survival pattern, we also considered chemotherapy and radiotherapy as additional variables. Accordingly, patients who received chemotherapy (66.5 ± 5.7 vs. 83.1 ± 2.5 months) or radiotherapy (58.8 ± 7.6 vs. 82.9 ± 2.4 months) both exhibited worse prognosis (Figure 3i,j, both *p* < 0.05). This may be caused by the fact that only patients with advanced stages are more willing to accept postoperative adjuvant therapies, which showed a disappointing prognosis.

**Figure 4:**
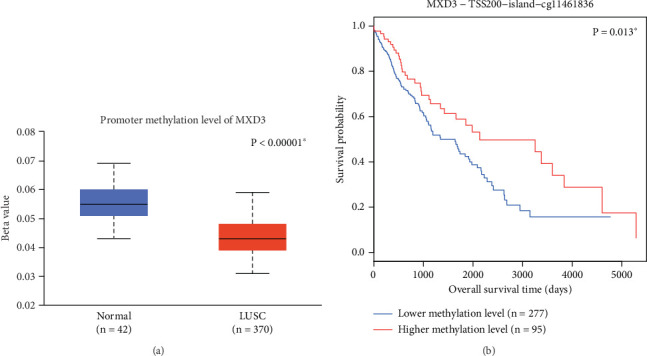
Methylation level of MXD3 in LUSC and its prognostic significance. (a) Based on the TCGA dataset, the methylation level of MXD3 in LUSC tissues is significantly lower than that in normal lung tissues. (b) Moreover, the lower methylation level of MXD3 is significantly correlated with worse overall survival of LUSC patients in the TCGA dataset.

Interestingly, our analysis of MXD3 methylation status, derived from the TCGA dataset, reveals significant findings regarding its epigenetic regulation in LUSC. Methylation levels of MXD3 were found to be significantly lower in LUSC tissues compared to normal lung tissues (Figure 4a, *p* < 0.001), aligning with the observed differences in MXD3 RNA expression presented in Figure 1c. Furthermore, our supplementary analysis demonstrated that lower methylation levels of MXD3 are associated with worse survival outcomes (Figure 4b, *p* = 0.013). This correlation suggests that methylation status not only influences MXD3 expression but may also serve as an important prognostic indicator in LUSC.

### 3.4. Multivariate Cox Regression Analysis for Cancer-Specific Survival in Enrolled LUSC Patients

Table 3 presents the results of the multivariate Cox regression analysis conducted to identify independent prognostic factors for cancer-specific survival in enrolled LUSC patients. The analysis included variables such as tumor size, T stage, N stage, radiotherapy, chemotherapy, and MXD3 expression level. After adjusting for potential confounding factors, MXD3 expression level emerged as a significant independent predictor of cancer-specific survival in LUSC patients (HR = 3.028, 95% CI: 1.245–7.361, ⁣^∗^*p* = 0.015). Patients with high MXD3 expression levels had over threefold increased risk of cancer-related mortality compared to those with low expression levels, underscoring the robust prognostic significance of MXD3 in LUSC.

N stage was also identified as an independent prognostic factor for cancer-specific survival, with a significantly increased HR (HR = 2.327, 95% CI: 1.364–3.970, ⁣^∗^*p* = 0.002). This finding highlights the importance of lymph node involvement as a predictor of poor prognosis in LUSC patients. Additionally, the T stage showed a significant association with cancer-specific survival (HR = 1.549, 95% CI: 1.008–2.380, ⁣^∗^*p* = 0.046), indicating its relevance in prognostic assessments.

Conversely, tumor size (HR = 1.141, 95% CI: 0.513–2.534, *p* = 0.747), radiotherapy (HR = 0.936, 95% CI: 0.269–3.258, *p* = 0.917), and chemotherapy (HR = 0.485, 95% CI: 0.149–1.583, *p* = 0.231) did not demonstrate significant associations with cancer-specific survival in the multivariate analysis (*p* > 0.05). These results suggest that while tumor size, radiotherapy, and chemotherapy may influence prognosis, their effects may be confounded by other variables such as lymph node involvement and MXD3 expression level. Overall, the multivariate Cox regression analysis reaffirms the independent prognostic value of MXD3 expression level and N stage in predicting cancer-specific survival outcomes in LUSC patients.

## 4. Discussion

LUSC represents a formidable challenge in oncology, characterized by its aggressive behavior and limited treatment options [11, 12]. In this study, we investigated the oncogenic role of MXD3 in LUSC and its implications for patient prognosis. Our findings shed light on the prognostic significance of MXD3 expression and its potential as a therapeutic target in LUSC management.

The observed heterogeneity in MXD3 expression among LUSC patients underscores its complex role in tumorigenesis. High MXD3 expression was associated with advanced tumor differentiation grade, larger tumor size, and advanced T and N stages, indicating a potential role of MXD3 in tumor aggressiveness and metastasis. These findings are consistent with previous studies implicating MXD3 dysregulation in various human malignancies, including leukemia, liver cancer, renal cancer, and glioma, highlighting the broad relevance of MXD3 in cancer biology [13–19].

Our survival analyses revealed that high MXD3 expression was significantly associated with shorter overall and cancer-specific survival in LUSC patients. These results are consistent with prior studies demonstrating the prognostic significance of MXD3 expression in other cancer types. For instance, Barisone et al. reported that MXD3 can significantly modulate glioma cell proliferation [20, 21].

Multivariate Cox regression analysis identified MXD3 expression level and lymph node involvement (N stage) as independent prognostic factors for cancer-specific survival in LUSC patients. These findings underscore the clinical relevance of MXD3 as a prognostic biomarker, offering valuable insights into risk stratification and treatment decision-making in LUSC. The identification of MXD3 as an independent prognostic factor complements existing prognostic markers and may facilitate more accurate patient prognosis and personalized treatment approaches.

While our study presents significant findings with a cohort of 199 patients, we recognize that a larger sample size could enhance the robustness of our conclusions. The relatively small number of participants may limit the generalizability of our results and introduce potential sampling biases. To strengthen our conclusions, we also compared MXD3 expression levels with data from the TCGA dataset, which included 486 LUSC samples. Our analysis revealed that MXD3 transcription levels are significantly elevated in LUSC tissues compared to adjacent normal lung tissues. This correlation between our clinical findings and the external dataset supports the relevance of MXD3 as a biomarker in LUSC. Furthermore, our study contributes to the growing body of evidence supporting MXD3 as a potential therapeutic target in cancer. Targeting MXD3 and its downstream signaling pathways may offer new avenues for the development of novel therapeutic interventions in LUSC. For example, Duong et al. reported the potential of targeted therapy for neuroblastoma by silencing the MXD3 gene using siRNA [22], suggesting MXD3 as a promising therapeutic target in breast cancer treatment [23].

Our investigation into the methylation status of MXD3 reveals significant findings regarding its role in LUSC. Analysis of the TCGA dataset indicates that MXD3 exhibits markedly lower methylation levels in LUSC tissues compared to adjacent normal lung tissues. This observation supports the hypothesis that epigenetic modifications may play a crucial role in regulating MXD3 expression. Additionally, our supplementary analysis demonstrated a correlation between decreased methylation levels of MXD3 and poorer survival outcomes. These results suggest that methylation status may serve as an important prognostic factor, reflecting the aggressive nature of tumors with reduced MXD3 expression. The findings highlight the potential of MXD3 as not only a biomarker for prognosis but also as a candidate for therapeutic targeting. Further research is warranted to elucidate the underlying mechanisms by which methylation affects MXD3 expression and its implications for tumor progression and treatment response in LUSC.

## 5. Conclusions

Our study provides compelling evidence for the oncogenic role of MXD3 in LUSC and its prognostic significance. MXD3 expression level emerges as a promising biomarker for risk stratification and treatment optimization in LUSC patients. Future studies elucidating the molecular mechanisms underlying MXD3-mediated tumorigenesis and exploring therapeutic strategies targeting MXD3 are warranted to advance our understanding and management of LUSC.

## Figures and Tables

**Table 1:** Clinicopathological features of enrolled LUSC cases and their associations with MXD3 level.

**Variable**	**Number**	**MXD3 level**		**p**
	**(** **n** = 199**)**	**Low (** **n** = 100**)**	**High (** **n** = 99**)**	
*Age (years)*				0.716
≤ 65 yrs	110	54	56	
> 65 yrs	89	46	43	
*Sex*				0.726
Female	105	54	51	
Male	94	46	48	
*Differentiation grade*				0.023⁣^∗^
Well	28	17	11	
Moderate	101	57	44	
Poor	70	26	44	
*Tumor site*				0.101
Upper lobe	123	69	54	
Middle lobe	14	5	9	
Lower lobe	62	26	36	
*Laterality*				0.514
Left lung	87	46	41	
Right lung	112	54	58	
*Tumor size*				0.006⁣^∗^
< 3.0 cm	108	64	44	
≥ 3.0 cm	91	36	55	
*T stage*				0.004⁣^∗^
T1	85	51	34	
T2	77	40	37	
T3	26	6	20	
T4	11	3	8	
*N stage*				< 0.001⁣^∗^
N0	144	87	57	
N1	24	4	20	
N2	31	9	22	
*Radiotherapy*				0.025⁣^∗^
No	170	91	79	
Yes	29	9	20	
*Chemotherapy*				0.013⁣^∗^
No	148	82	66	
Yes	51	18	33	

⁣^∗^*P* < 0.05.

**Table 2:** Univariate analyses for the cancer-specific survival of enrolled LUSC patients.

**Variable**	**Number**	**Cancer-specific survival**		**p**
	**(** **n** = 199**)**	**M** **e** **a** **n** ± **SD (months)**	**5-year (%)**	
*Age (years)*				0.093
≤ 65 years	110	82.5 ± 2.7	84.7%	
> 65 years	89	74.6 ± 4.2	71.5%	
*Sex*				0.961
Female	105	77.1 ± 3.4	79.2%	
Male	94	80.5 ± 3.4	78.9%	
*Differentiation grade*				0.078
Well	28	84.6 ± 4.3	89.4%	
Moderate	101	79.6 ± 3.0	82.0%	
Poor	70	73.7 ± 4.8	69.2%	
*Tumor site*				0.349
Upper lobe	123	81.6 ± 3.1	81.4%	
Middle lobe	14	65.9 ± 9.8	60.5%	
Lower lobe	62	76.4 ± 4.2	77.3%	
*Laterality*				0.351
Left lung	87	82.9 ± 3.2	82.4%	
Right lung	112	73.7 ± 3.3	75.6%	
*Tumor size*				0.047⁣^∗^
< 3.0 cm	108	82.6 ± 2.8	83.2%	
≥ 3.0 cm	91	74.4 ± 4.3	73.8%	
*T stage*				< 0.001⁣^∗^
T1	85	84.8 ± 2.9	85.3%	
T2	77	82.2 ± 3.6	81.3%	
T3	26	51.7 ± 6.9	52.8%	
T4	11	57.8 ± 7.6	77.8%	
*N stage*				< 0.001⁣^∗^
N0	144	84.6 ± 2.2	86.1%	
N1	24	56.6 ± 6.1	61.4%	
N2	31	60.6 ± 8.5	53.5%	
*Radiotherapy*				0.004⁣^∗^
No	170	82.9 ± 2.4	81.8%	
Yes	29	58.8 ± 7.6	59.2%	
*Chemotherapy*				0.031⁣^∗^
No	148	83.1 ± 2.5	82.3%	
Yes	51	66.5 ± 5.7	68.5%	
*MXD3 level*				< 0.001⁣^∗^
Low	100	89.1 ± 2.1	92.5%	
High	99	64.9 ± 3.9	61.7%	

⁣^∗^*P* < 0.05.

**Table 3:** Multivariate analysis for the cancer-specific survival of enrolled LUSC patients.

**Variable**	**Hazard ratio**	**95% confidence interval**	**p**
Tumor size	1.141	0.513–2.534	0.747
T stage	1.549	1.008–2.380	0.046⁣^∗^
N stage	2.327	1.364–3.970	0.002⁣^∗^
Radiotherapy	0.936	0.269–3.258	0.917
Chemotherapy	0.485	0.149–1.583	0.231
MXD3 level	3.028	1.245–7.361	0.015⁣^∗^

⁣^∗^*P* < 0.05.

## Data Availability

The data that support the findings of this study are available from the corresponding author upon reasonable request.
